# Inference generation in older adults: Comparing pictorial and textual comprehension in the context of cognitive decline

**DOI:** 10.3758/s13421-025-01736-7

**Published:** 2025-07-18

**Authors:** Ekaterina Varkentin, Irina R. Brich, Ulrike Sünkel, Anna-Katharina von Thaler, Gerhard W. Eschweiler, Markus Huff

**Affiliations:** 1 Perception and Action Lab, Leibniz-Institut für Wissensmedien, Schleichstraße 6, 72076 Tübingen, Germany; 2 Tübingen Center for Mental Health (TüCMH), Department of Psychiatry and Psychotherapy, Tübingen University Hospital, Tübingen, Germany; 3 German Center for Mental Health (DZPG), Partner Site Tübingen, Tübingen, Germany; 4 Department of Neurology, Kiel University, University Medical Centre Schleswig-Holstein, Kiel, Germany; 5 Department of Neurodegeneration, Hertie Institute for Clinical Brain Research, University of Tübingen, Tübingen, Germany; 6 Geriatric Center, Tübingen University Hospital, Tübingen, Germany; 7 Department of Psychology, University of Tübingen, Tübingen, Germany

**Keywords:** Narrative comprehension, Inferencing, Age, Codality, Risk factors, Preventive factors

## Abstract

Narrative comprehension, a cognitive skill essential for social participation, relies on a range of abilities, including memory and inference-making. While aging-related cognitive changes are well-documented, research on narrative comprehension in aging populations yields mixed findings, underscoring the importance of this study. This preregistered study examines how age, education, and presentation format (i.e., the narrative’s codality: pictorial vs. textual) influence inference generation in older adults (*N* = 143, ages 62–86 years). Participants were presented with pictorial and textual stories consisting of three panels, with the second panel replaced with a blank panel. Their task was to comprehend the stories and determine whether the inferences provided for the missing event were correct or incorrect. Results reveal that pictorial narratives were better comprehended than textual ones; however, this advantage attenuates with increasing age. Contrary to expectations, narrative comprehension was largely resilient to age-related declines, as neither age nor education significantly impaired performance. Exploratory analyses tested the influence of protective (e.g., physical and mental activity, companionship) and risk factors (e.g., depression, anxiety, chronic pain, stress, and poor sleep) but found no significant impact on comprehension. Notably, narrative comprehension correlated with memory performance but not with other cognitive abilities, underscoring its specificity within the broader cognitive domain. These findings highlight the stability of narrative comprehension across media and aging, while also suggesting a narrowing pictorial advantage with age. Implications for existing cognitive theories and future research directions are discussed.

**Supplementary Information:**

The online version contains supplementary material available at 10.3758/s13421-025-01736-7.

Media and narrative comprehension research is currently at a pivotal stage, with an increasing number of studies examining the comprehension of visual narratives. Although the literature comparing text-based and picture-based story comprehension is expanding, much of this research has concentrated on children’s reading development (Kendeou et al., 2016). Narrative comprehension involves interrelated cognitive skills such as text integration, metacognitive monitoring, inferencing, and working memory (Hwang et al., 2024; Lynch et al., 2008; Oakhill et al., 2003). Given that those cognitive skills evolve and mature with age, it stands to reason that narrative comprehension should also develop across the lifespan. Whereas the development of narrative comprehension in children is described in detail in recent research (e.g., Cohn, 2019), little is known about how visual narrative comprehension and inference generation, as components of narrative comprehension, develop in older people. However, the skill to comprehend narratives and draw inferences is especially crucial for older people facing the increasingly difficult challenge of staying connected to society and keeping up to date in a rapidly changing world. Many cognitive skills undergo noticeable changes in the later years of one’s life (Brayne et al., 1995; Stine-Morrow & Radvansky, 2017) and being an important part of cognition, the fundamental research question is whether narrative comprehension remains stable even in older age. The present study addresses this question by exploring inferencing processes in visual narrative comprehension using a sample of participants ages 62 and older.

## Building a mental model

Theories of comprehension universally assume that comprehension is grounded in building a mental model (Kintsch, 1988). Mental models are internal cognitive representations that readers construct to understand and retain the information presented in a text. It involves creating a mental image of the described situation, including the various entities, their interactions, locations, and other relevant details in the text (Kintsch, 1988; Zwaan & Radvansky, 1998). This model suggests that during reading or listening, individuals first build propositional representations based on the text. Then, through integration, these propositions are organized into a coherent structure that reflects the underlying meaning or “situation model” of the discourse (Kintsch, 1988). This model helps readers make sense of the text, remember its content, and draw inferences about the relationships between different elements in the narrative (Huff et al., 2018). One influential theory describing the phases of building mental models is the structure-building framework (Gernsbacher et al., 1990).

Gernsbacher et al. (1990) specified the phases of constructing mental models in the structure-building framework. It describes the comprehension process as the creation of a cohesive mental representation of incoming information through sequential steps: laying a foundation, followed by mapping new information, and, if necessary, shifting to a new foundation. Laying a foundation involves establishing an initial mental structure that encapsulates the information presented in a scene. Mapping occurs when incoming information is related to the existing structure and can be integrated into it. Shifting happens when new information is inconsistent or unrelated to the current structure, requiring the creation of a new mental substructure to accommodate it (Gernsbacher, 1997; Gernsbacher et al., 1990). In the following paragraph, we discuss how these cognitive processes operate across different presentation formats, such as pictorial and textual.

### Visual and textual narratives

Narrative information can be conveyed as text (Magliano et al., 2016; Radvansky & Copeland, 2010), picture stories (Cohn, 2020; Huff et al., 2020, 2025), audio (Huff et al., 2018), or video (Huff et al., 2014). The structure-building framework posits that comprehension processes operate independently of visual and auditory modalities^1^ (Gernsbacher et al., 1990). Further, Gernsbacher and Faust (1991) introduced the concept of a *general comprehension skill*, the ability to comprehend linguistic and nonlinguistic information from different modalities. According to this concept, the processes responsible for comprehension are comparable across different codalities (i.e., text and picture stories). Kendeou and colleagues (Kendeou et al., 2016) argue that inference skill is a general skill that is media independent. This raises the question of whether this would be the case for older adults.

However, recent evidence showed that pictorial narratives are generally better understood than textual ones across different education and age groups in a representative German sample (Huff et al., 2025). Furthermore, processing narrative texts requires more time than narrative picture stories, reflected in longer viewing times for texts compared with pictures (Huff et al., 2020). According to the structure-building framework (Gernsbacher, 1997; Gernsbacher et al., 1990), processes such as laying a foundation, mapping, and shifting may demand less cognitive effort in the context of pictorial narratives. In this way, the theoretical and empirical yielded research mixed findings. However, much of the existing literature highlights similarities in comprehension across narrative codalities rather than fundamental differences (e.g., Gernsbacher et al., 1990; Magliano et al., 2012; Meitz et al., 2020). A lack of empirical studies directly comparing comprehension across codalities and memory skills remains, particularly in older populations. This theoretical foundation sets the stage for a deeper examination of the cognitive processes involved in narrative comprehension, particularly in distinguishing how front-end and back-end processes operate across different codalities and age groups.

### Front-end and back-end processes during comprehension

Although the structure-building framework does not specify which processes (such as attention or working memory) are different in poor and good comprehenders, more recent accounts, such as the scene perception and event comprehension theory (SPECT; Loschky et al., 2020) and the front-end back-end framework (Magliano et al., 2013) offer a nuanced approach to understanding comprehension differences by distinguishing between front-end and back-end processes in visual narrative comprehension. Front-end processes include attentional selection, which determines the information to be processed, and information extraction, ensuring the accurate retrieval of meaningful content from pictures. According to Magliano et al. (2013), front-end processes lead to the computation of the event model representing the “now” moment in a narrative. The event model will then be developed by the back-end processes to comprehend the dynamic structure of the narrative. Back-end processes are tasked with constructing and sustaining the working memory representation of the current event model and are associated with both working and long-term memory. Notably, these models are intricately linked with event models stored in episodic long-term memory, preexisting schemata, and executive functions, as outlined by Loschky et al. (2020). Front-end and back-end processes function somewhat differently across modalities. For example, Magliano et al. (2013) argue that while front-end processes differ between visual and textual modalities, back-end processes remain the same. While orthographic, phonological, lexical-syntactic, and semantic processing occur across all modalities, gist processing (e.g., recognizing locations of events), object processing, and motion processing are specific to visual or graphic modalities.

SPECT (Loschky et al., 2020) and the front-end–back-end framework (Magliano et al., 2013) suggest two potential reasons for impaired narrative comprehension in higher age: first, a possible inefficiency in attentional selection, and second, a malfunction in the working memory. Considerable evidence shows that working memory capacity is associated with the ability to generate complex inferences (Linderholm, 2002; Rai et al., 2011; St George et al., 1997). Given that studies, like those by Chen et al. (2003), Jost et al. (2011), and Myerson et al. (2003), indicate that older adults often experience a decline in working memory capacity, SPECT becomes particularly pertinent when examining this demographic. The critical question then becomes: Is a potential decline in narrative comprehension directly related to the usually observed decrease in working memory, or is it a distinct capability that remains resilient to age-associated alterations?

### Bridging inferences

As linking consecutive parts of the story is essential for the comprehension of the narrative, bridging inference generation emerges as a fundamental process in narrative comprehension (Graesser et al., 1994). Generating bridging inferences—one of the primary processes supporting mapping in terms of the structure-building framework—is based on the attempt of readers to construct the meaning of events (Graesser et al., 1994). Magliano et al. (2016) propose that the semantic overlap between two consecutive frames of a pictorial narrative is crucial for initiating inference generation processes. Only a moderate overlap activates inference generation (Magliano et al., 2016)—for example, when two images involve the same characters and surroundings but depict different actions and/or emotions.

Importantly, cognitive processes can function differently with increasing age. In the next paragraph, we highlight the relevant findings regarding aging and narrative comprehension.

### Aging and narrative comprehension

Several studies investigated comprehension in age or underlying cognitive processes like working memory, attention, construction, and maintenance of situation models. In their review of discourse processing across the lifespan, Stine-Morrow and Radvansky (2017) indicate that findings on age-related comprehension are mixed. They conclude that age-related declines in processing speed, working memory capacity, reasoning, executive control, and other fluid abilities are typical (Salthouse, 2010; Stine-Morrow & Radvansky, 2017). At the same time, knowledge-based processes, including acquired skills, semantic memory, and crystallized verbal abilities (e.g., vocabulary), are generally well preserved in older adults (at least into the eighth decade of life; Baltes, 1997; Li et al., 2004; Stine-Morrow & Radvansky, 2017).

Research on listening comprehension demonstrated its general stability until the age of 65 to 70 years but a significant decline after the age of 70 years (Sommers et al., 2011). However, Cohen (1987) reported age differences in bridging inference generation during speech comprehension. A study examining textual inferences found that both younger and older adults can draw causal bridging and predictive inferences when reading multiple sentences (Valencia-Laver & Light, 2000). Research on predictive inference generation using pairs of sentences, each made up of a predicting sentence and its control, suggests that older adults can make inferences to the same extent as college-age adults (McKoon & Ratcliff, 2013). This study was particularly concerned with predictive inferences that might link an event with its outcome. However, there remains ambiguity regarding how visual narrative comprehension operates within the older sample.

Magliano et al. (2012) examined the impact of story modality on event segmentation, a fundamental component of event comprehension, in both older adults (ages 62 to 92) and younger adults (ages 18 to 22). They reported similar levels of segmentation agreement between older and younger adults in both textual and pictorial stories. Older adults use situation-level information for segmentation in a manner similar to younger adults (Magliano et al., 2012). While research on narrative comprehension across different age groups exists, the findings are often mixed. Further research is needed to explore narrative comprehension more thoroughly, particularly to understand how the inferencing process functions in visual narratives in older adults.

In addition to typical age-related cognitive changes, such as slower processing speed, reduced working memory, and weaker executive function, other factors can also influence narrative comprehension. Stress, pain, and mental health conditions like depression or mild cognitive impairment have been shown to negatively affect cognitive performance (Bell et al., 2022; Weisenbach et al., 2012). These conditions become more common with age. Kircanski et al. (2012) found that depressed individuals may use less effective memory strategies. Bell et al. (2022) found in their recent longitudinal study that pain at baseline was related to worse baseline memory performance and a decline in processing speed. Nearly half of older adults also report difficulties with attention and reasoning, and worse cognitive status due to pain. Further, many global changes in recent years, such as the COVID-19 pandemic, wars, and climate change, can increase the stress level people are exposed to in everyday life and contribute to rising depression rates (Hammen, 2005). Fortunately, research has shown that cognitive decline is not inevitable and can be mitigated through social engagement, intellectual stimulation, and physical activity (Butler et al., 2004). However, applying these findings on how factors like depression, pain, and stress affect cognition to the specific domain of narrative comprehension in the older subsample is not straightforward. For example, a study representative of education and age in Germany showed that age does not seem to play an important role in narrative comprehension, measured with the bridging inference generation test, while education does (Huff et al., 2025). It did not, however, consider how specific risk factors like depression or anxiety might play a role. Thus, to fully understand narrative comprehension in older adults, research must link narrative comprehension and preventive (positive association) and risk factors (negative association). The present study fills this gap by testing a sample of older adults (>70 years) with the bridging inference generation measurement in relation to various protective factors, such as regular physical activity, mental fitness, and stable social connections, as well as risk factors, including depression, anxiety, chronic pain, long-term stress, and sleep quality.

### The present study

The present study consists of two parts: an experiment with participants (*N* = 143) of a longitudinal aging study (part one) and an in-depth analysis of this data with variables collected in the longitudinal study (part two). In the experimental first part, we investigate narrative comprehension by testing bridging inference generation—that is, inferences generated to bridge gaps in the narration (Huff et al., 2020, 2025; Magliano et al., 2016). By measuring this process, we can draw conclusions about overall comprehension skills. Participants were presented with textual and visual narratives with a missing bridging event to assess narrative comprehension. Bridging events in comics/picture stories are panels depicting information that bridges the narrative gap between two elements (panels). Deleting bridging event information and depicting only the beginning and end state (Huff et al., 2020; Magliano et al., 2016) is an established experimental procedure to research inference generation and, thus, narrative comprehension. If a bridging event is missing in a visual or textual story, it must be inferred from the information presented in the beginning and end states. This process consumes cognitive resources that can be measured in different ways—namely, as an increase in viewing time for the subsequent panel (Huff et al., 2020; Magliano et al., 2016, 2017), surface information recognition, or answering comprehension questions (Gernsbacher et al., 1990; Huff et al., 2025). Our study tests the formation of inferences between the beginning and end states. After each story, participants receive one statement either describing the correct inference for the missing bridging event or a false inference to be judged for correctness.

The experiment investigates how narrative comprehension is influenced by education, age, and codality. For this experiment, we derived two hypotheses (preregistered here: https://aspredicted.org/897_7QW). We expected narrative comprehension to increase with higher education (H1), to decrease with higher age (H2), and we expected narrative comprehension to be higher for pictorial than for textual narratives independent of education or age (no interaction of education and age effects with story codality; H3). Exploratorily, we also investigated the relationship between cognitive skills and narrative comprehension.

## Methods

### Transparency and openness

#### Transparency in data, analysis, and materials

We affirm that the de-identified data on which the study conclusions are based are available at the project’s Open Science Framework page (https://osf.io/2zyuv/).

We affirm that the syntaxes used to conduct analyses in a statistical package R are available at the project’s Open Science Framework page (https://osf.io/2zyuv/). For data analysis we used R (Version 4.3.1) (R Core Team, 2023) and RStudio (Version 2023.12.0) (RStudio Team, 2021). We used following packages for R: *stringr* (Version: 1.5.0; Wickham, 2022), *sciplot* (Version 1.2-0; Morales et al., 2020), *lme4* (Version 1.1–33; Bates et al., 2015), *lattice* (Version: 0.21–8; Sarkar, 2008), *ggplot2* (Version 3.4-2; Wickham, 2016), *lmerTest* (Version 3.1-3; Kuznetsova et al., 2017), *car* (Version: 3.1-2; Fox & Weisberg, 2019), *sjPlot* (Version: 2.8.15; Lüdecke, 2023), *Hmisc* (Version: 5.1-0; Harrell, 2023), *dplyr* (Version: 1.1.2; Wickham et al., 2023), *ggpubr* (Version: 0.6.0; Kassambara, 2023), *tidyverse* (Version: 2.0.0; Wickham et al., 2019), *broom.mixed* (Version: 0.2.9.4; Bolker & Robinson, 2022), and *jtools* (Version: 2.2.2; Long, 2022).

**Fig. 1:**
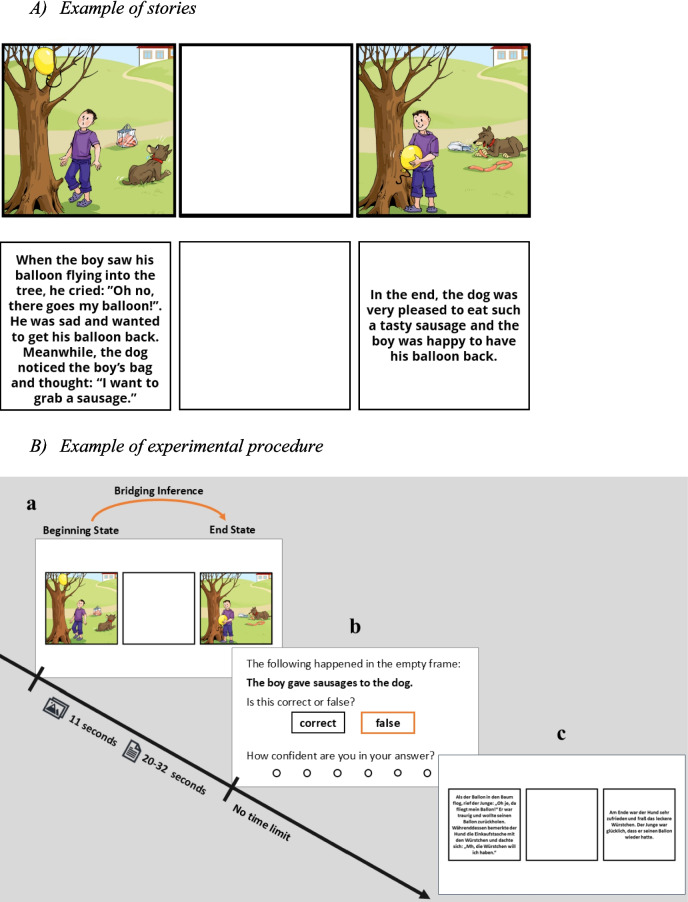
**A)** Example of the used pictorial and textual stories generated from the MAIN; **B) **Examples of the experimental procedure. **a** The measurement of the bridging inference generation in the pictorial codality. **b** The response panel. **c** The measurement of the bridging inference generation in the textual codality. (Color figure online)

We present an example of the study materials (see Fig. 1A). The license of the underlying material prevents its publication in this study as it is part of a diagnostic tool (Multilingual Assessment Instrument for Narratives [MAIN]; Gagarina et al., 2019; https://main.leibniz-zas.de/). Nevertheless, all visual materials employed in this study can be retrieved from the website (https://main.leibniz-zas.de). Access to these materials requires prior acceptance of the corresponding copyright and license agreement.

#### Transparency in research design

We affirm that we report how we determined our sample size, all data exclusions, all manipulations, and all measures in the Participants and Materials sections. If specific exclusions were required due to missing data in particular values, we have documented such occurrences in the Results section.

### Preregistration

Both parts of the study (including study design, hypotheses, and analytic plan) were preregistered (reanalysis of TREND data: https://osf.io/4kf3e?view_only=6248cb53a51242e48a9c6774def2d40e; narrative comprehension assessment in TREND sample: https://aspredicted.org/897_7QW).

### Participants

In collaboration with the University Hospital for Psychiatry and Psychotherapy Tübingen (Germany) and the TREND study (www.trend-studie.de, **T**uebingen evaluation of **R**isk factors for **E**arly detection of **N**euro**D**egeneration), we tested 143 participants (*N* = 143, *M* = 72.06 years, range 62–86 years, *SD* = 5.36, 62 women, 81 men). We are unable to provide the racial distribution of the sample, as questions related to ethnic background are not included in the studies conducted in Germany due to legal restrictions. The TREND study received ethical approval by the University Hospital Tübingen ethics committee. Additionally, the experimental first part was approved by the local ethics commission of the Leibniz Institut für Wissensmedien (IWM). The experimental part of the study took place in January–February 2022. The participants, who indicated their ability to participate on a personal computer or notebook, were contacted via e-mail requesting participation in the present experiment. We included the data of all participants who participated within 4 weeks. The original dataset consisted of 143 participants. We excluded three participants who reported not having seen the stories properly in the allotted time. Further, one participant was excluded because of a completion time over 60 min. We additionally excluded two participants because of double participation. Further exclusion criteria were Alzheimer’s dementia, dementia, Parkinson’s disease, and mild cognitive impairment according to ICD-10 criteria. No participant had to be excluded based on these criteria. The final data included 137 participants (60 women, 77 men). The average age in the final subsample was *M* = 71.92 years, *SD* = 5.24, with an age range of 62 to 86 years.

### Material

We used the narratives of the MAIN (Multilingual Assessment Instrument for Narratives; Gagarina et al., 2019) to test narrative comprehension. MAIN is a set of picture and text stories designed to test bilingual children’s narrative skills and language impairments (Fig. 1A). It consists of four stories, each depicting three storylines within a six-picture sequence based on a multidimensional model of story organization and on the linguistic structure of a story. The stories are controlled for cognitive and linguistic complexity, parallelism in macrostructure and microstructure, as well as cultural appropriateness and robustness (Gagarina et al., 2019). We adapted the material for our bridging inference generation test, which resulted in 12 picture stories, each consisting of three pictures: beginning state, bridging event, and end state (see example in Fig. 1A; Huff et al., 2025). The same procedure was adapted to the text versions of the stories.

For presentation, the pictures were shown simultaneously in a row, while an empty frame replaced the middle picture (bridging event) of each story (see Fig. 1). We generated and selected the false and correct text inferences based on the content of the bridging event. False and correct inferences are constructed as a single sentence that states the main event happening in the bridging event (i.e., “the bird attacked the fox”). Each participant was presented with eight of the 12 stories. Each story was paired with either a true or a false inference statement regarding the blank frame. Finally, each participant received two false and two true inferences for the four textual and four pictorial story clips. The selection of the eight stories, their codality (text/picture), and the combination with true/false inferences was counterbalanced across participants (one of the 256 possible combinations was randomly selected for each participant; Huff et al., (Huff et al., 2025)). Codality of the stories was manipulated within-subjects.

### Procedure for collecting narrative comprehension data

We conducted the study online using SoSciSurvey. The experiment was in German and began with providing general information about the study goals. As the informed consent of the TREND study was also applicable to the current study, participants were asked if they wanted to proceed with the current study by clicking on “next” (data retraction was also possible through contacting the TREND study team). They were instructed to close the browser if they did not want to proceed with this study. Afterward, we collected the TREND IDs, the unique identifiers each participant was assigned in the TREND study. This step was necessary to match the results of our narrative comprehension measurement with the longitudinal data from the TREND study. Demographic data, including age, gender, and education, was collected on the next page, and could also be obtained from the TREND dataset. In addition, participants provided information about their experience with reading text and picture stories in their adolescence and currently. This information was assessed with questions such as “How often did you read visual/textural stories such as . . . during your childhood and youth/nowadays?” and 7-point Likert scale (from *never* to *always*). After that, participants estimated statements “I can read texts well” and “I find it difficult to read texts” with the help of a 6-point scale (from *fully disagree* to *fully agree*). The reading experience variables were not analyzed in this study.

Next, participants worked on the narrative comprehension task involving a bridging-inference generation test (Huff et al., 2025; see Fig. 1B). We instructed the participants that they would see a couple of picture/text stories with the middle part of the story missing that they needed to comprehend. After each story, they were asked to indicate whether a presented inference for the missing bridging event was correct or wrong. Four practice trials with feedback (two text and two picture stories) demonstrating the experimental procedure preceded the main experiment to ensure participants understood the task. Participants viewed each pictorial story for 11 s and each textual story dependent on the number of words calculated at an average reading time of 175 words per minute (Brysbaert, 2019) plus 5 s for the blank panel. Upon time expiry, participants were automatically presented with either a correct or a false inference statement in text form. They indicated whether this statement is correct or false. In addition, they were asked to rate their confidence (from very unsure to very sure) in the bridging inference task (the confidence measure was not analyzed in this study). Figure 1A demonstrates the example of the used pictorial and textual stories generated from the MAIN (Gagarina et al., 2019), and Fig. 1B illustrates the experimental procedure.

After finishing the bridging-inference generation test for the eight stories, participants were asked whether they could read most of the stories in the given time and whether they could sufficiently see the text or pictures for most of the stories.

### Education and age

To cover educational classifications by the ISCED 1997 (Organisation for Economic Co-operation and Development [OECD], 1999; Simonson & Hameister, 2017) for the German education system, we generated two questions for assessment of educational background. The first question covered school education, with the following answering options: “still a student,” “finished school without degree,” “Haupt-/Volksschulabschluss, […]” (9 years of schooling), “Realschulabschluss […]” (10 years of schooling), “Fachhochschulreife […]” (approx. 11–12 years of schooling and a practical education period), “Abitur […]” (12–13 years of schooling). Participants were asked to choose their highest school education. The second question covered professional degree or training qualification, with the options: “no professional degree,” “Abschluss einer Lehre […]” (finished apprenticeship), “Abschluss an einer Fachschule […]” (graduation from a professional school), “Beamtenausbildung für den einfachen oder mittleren Dienst” (training for civil servants low- to middle-level), “Beamtenausbildung für den gehobenen Dienst” (training for civil servants for higher service), “Hochschul- oder Universitätsabschluss […] “(University degree), “Promotion oder Habilitation” (PhD or habilitation). Participants were asked again to state their highest qualification. Both questions offered an option to enter free text (these answers were recategorized to fit the offered categories). Building on prior research concerning narrative comprehension and education by Huff et al. (2025), we categorized the “education” variable into three groups: low (answer options 1–3), middle (specifically the “Realschulabschluss” option), and high (all other answer options). This categorization was adopted because the question on school education has been shown to be a reliable metric. We had following distribution in our sample: low (*N* = 27), middle (*N* = 28), and high (*N* = 82). We did not take into account professional education or training qualifications.

The age variable was treated as a continuous variable, which was centered before the statistical analysis.

### TREND data/variables

Data about cognitive skills, as well as protective and risk factors, were received from the longitudinal TREND study. The study was launched in 2009 and tests 1,201 individuals over 50 years. Participants are tested biennially with a comprehensive, mainly quantitative assessment battery. It includes different examinations – for example, neurological, cognitive, motor examinations, medical history, brain sonography, and filling out different questionnaires. We received longitudinal data from all assessment points for each of the participants, as well as the latest data for cognitive skills and for the protective and risk factors, which are listed below.

### Cognitive skills

To test the relationship between narrative comprehension and cognitive skills, we exploratively included results of cognitive tests from the TREND study in the analysis: the CERAD-plus test battery (Aebi, 2002; Ehrensperger et al., 2010; Morris et al., 1988, 1993) and the Montreal Cognitive Assessment (MoCA; Nasreddine et al., 2005) screening test. We included the total scores of the CERAD-plus and MoCA tests. Additionally, we incorporated the findings from two CERAD memory composite scores: Memory Total Score and Memory Index, as per Paajanen et al. (2014). The Memory Total Score was calculated by summing performance across three subtests:

1. *Word List Recall*, which involves three immediate recall trials of 10 unrelated words followed by a delayed recall;
2. *Word List Recognition*, measured by the sum of correctly recognized target and distractor words;
3. *Constructional Praxis Recall*, which includes a free recall of previously drawn figures.

The Memory Index was formed by calculating the average percentage of the retention scores on the *Word List Recall* and *Constructional Praxis Recall*, and *Word List Recognition* discriminability (Paajanen et al., 2014). Both the Memory Total Score and Memory Index were devised to offer a more precise and current evaluation of certain age-related conditions, such as prodromal Alzheimer’s disease and the progression of MCI (mild cognitive impairment; Dubois et al., 2007; Paajanen et al., 2014). They specifically measure delayed episodic memory performance.

These composite scores are deemed more reliable because they amalgamate the outcomes of multiple memory-related tasks from the CERAD test battery rather than the result of a singular task. Relying solely on individual memory tasks can be less reliable; for instance, even healthy older participants may exhibit subpar performance in at least one task (observed in roughly 30% of cases) (Paajanen et al., 2014). Notably, the Memory Total Score has demonstrated significant sensitivity in identifying participants with prodromal Alzheimer’s disease (Paajanen et al., 2014). Therefore, it is plausible to suggest that the Memory Total Score ranks as one of the most sensitive CERAD memory scales for older adults.

### Risk factors

#### Depression

The risk factor depression was measured using the Beck Depression Inventory scale (BDI-II; Beck et al., 1996; Hautzinger et al., 2009).

#### Anxiety

The anxiety risk factor was assessed through two questions in the survey. Participants marked yes or no to indicate the presence of anxiety disorder based on both the doctor’s assessment and their own self-assessment.

#### Chronic pain

Chronic pain was also assessed through two questions in the survey. Participants marked yes or no to indicate the presence of chronic pain based on both the doctor’s assessment and their own self-assessment.

#### Long-term stress

Long-term stress was measured using the Perceived Stress Scale (PSS; Cohen et al., 1983).

#### Sleep quality

We preregistered the variable “sleep quality” to be assessed as a combination of Item 16 from the BDI-II (change in sleeping habits) and Item 9 from the REM Sleep Behavior Disorder Screening Questionnaire (RBDSQ; Stiasny-Kolster et al., 2007). At the end, we only included Item 16 from the BDI-II as only a small number of participants completed the RBDSQ questionnaire. Item 16 from BDI-II has levels 0 (my sleeping habits have not changed), 1 (I sleep a little more/less than usual), 2 (I sleep a lot more/less than usual), and 3 (I sleep most of the day/I wake up 1–2 hours earlier than usual and then I can’t go back to sleep).

### Protective factors

#### Physical activity

The group of protective factors includes, firstly, physical activity, which we originally planned to assess with a combination of 10 yes/no questions about specific sports (e.g., jogging, swimming, cycling), a question about the overall frequency of sports activities (5-point scale from 1 = *no sport at all* to 5 = *>4 h/week* ; Thefeld et al., 1999), and a question that assesses how difficult is to climb stairs (with three levels). Finally, we used only the question about the overall frequency of sports activities, because otherwise we would lose too many participants due to the missing data. The overall frequency question represents a reliable measure of physical activity as it basically includes all other listed activities.

#### Mental fitness

The second possible protective factor for narrative comprehension was mental fitness. Originally it was planned to assess mental fitness with 11 yes/no questions about specific kinds of mental fitness (e.g., sudoku, crossword, reading) and with a question about the overall frequency of mental fitness activities (5-point scale from 1 = *no mental fitness activities at all* to 5 = *>4 h/week*). We only used the data of the question about the overall frequency of mental fitness because otherwise we would lose too many participants due to missing data. Nevertheless, the overall frequency question represents a reliable measure of mental fitness as it includes all other listed activities.

#### Stable social contacts (companionship)

The last protective factor, *stable social contacts (companionship)*, was assessed using the six-item loneliness scale (Gierveld & Tilburg, 2006). We used the reversed loneliness scale, assuming that lower loneliness means higher companionship and thus more stable social contacts.

## Results

The results will be structured as follows. We first present the results of an initial evaluation of how cognitive abilities and memory change with age. We then present the results of an analysis of the relationship between narrative comprehension and education (frequentist and Bayesian analyses), followed by an analysis of the relationship between narrative comprehension and age (frequentist and Bayesian analyses). Similar to the analyses used in Huff et al. (2025), for the frequentist analysis, the binomial narrative comprehension variable was analyzed with generalized linear mixed-effects models of the logit-family (“glmer,” *lme4* package; Bates et al., 2015). Each fitted model contained random intercepts for participants and stories. Fixed effects for the analyzed variables and their interaction were sequentially added to the intercept model and compared stepwise for model fit (likelihood-ratio test). We adhered to Jeffreys’s (1961) guidelines, where Bayes factors (BFs) of 1–3 are considered anecdotal, 3–10 substantial, 10–30 strong, 30–100 very strong, and values exceeding 100 indicate extreme support for the alternative hypothesis. Conversely, fractional values (such as 1/3 or 1/10) reflect comparable levels of evidence favoring the null hypothesis. For more details, including posterior predictive checks, see Appendix C.

Next, we present an explorative analysis evaluating relations between narrative comprehension and the MoCA and CERAD-plus cognitive tests in the context of different codalities. We refer to the Appendix B, where we present analysis of the connections between narrative comprehension and protective and risk factors.

For the analyses involving TREND data, an additional participant had to be excluded as the stated TREND-ID had no match in the TREND database.

### Age-related changes in memory and cognition

**Fig. 2:**
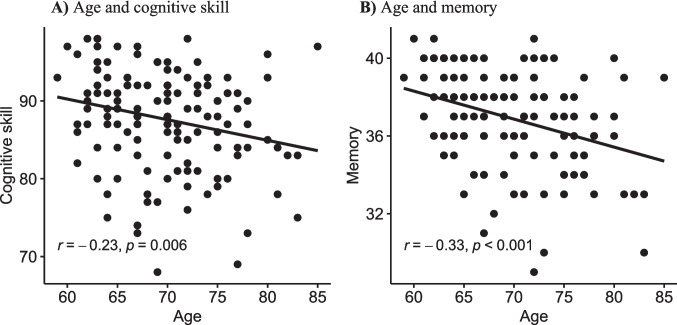
**A) **correlation between age and cognitive skills. B**)** correlation between age and memory

We calculated separate correlations between the factor age (at the time-point of latest TREND data collection for each variable) and results of the cognitive and memory test. For the Memory Total Score, one additional participant had to be excluded due to missing data. First, we observed a statistically significant decrease in general cognitive skills with increasing age, as assessed by the CERAD-plus test, *r* = −.23, *t*(134) = −2.79, *p* = .006 (see Fig. 2A). Secondly, a markedly significant decrease was noted in the Memory Total Score, a subdomain of the CERAD-plus test. Participants showed lower performance in Memory Total Score with increasing age *r* = −. 33, *t*(133) = −4.06, *p* < .001 (see Fig. 2B).

## Narrative comprehension, education and codality

### Frequentist analysis

Narrative comprehension was measured in terms of participants’ responses to the bridging inference statements being either true or false. We fitted generalized linear mixed-effects models for the dependent measure narrative comprehension and random intercepts for participant and story with the “glmer” function of the *lme4* package (Bates et al., 2015) and conducted stepwise model comparisons for each of the expected effects.

Starting from an intercept model, the fixed effects of education, codality, and their interaction were sequentially added and compared. We preregistered to control for mental disorders (for example, depression); however, due to the absence of clinically relevant levels of depression, we ultimately did not implement it.

We expected narrative comprehension to increase with higher education (H1). In contrast to Hypothesis 1, adding the *education* effect to the intercept model did not increase model fit, thus narrative comprehension did not increase with higher *education*, χ^2^(2) = 2.00, *p* = .368. Adding codality significantly improved model fit, χ^2^(1) = 4.25, *p* = .039. There was no interaction of *education* and *codality*, χ^2^(2) = 1.18, *p* = .555 (Table 1).

**Table 1:** Summary of the linear mixed model for the effects of education × codality

	Narrative comprehension		
*Predictors*	Odds ratios	CI	*p*
(Intercept)	2.62	1.57, 4.37	<.001
Education [middle]	1.52	0.81, 2.84	.193
Education [high]	1.55	0.94, 2.56	.087
Codality [picture]	2.40	1.07, 5.38	.033
Education [middle] × Codality [picture]	0.76	0.28, 2.09	.600
Education [high] × Codality [picture]	0.64	0.29, 1.44	.282

### Bayesian analysis

We used Bayesian analyses to complement the results of the frequentist analysis. We fitted the same models (including education and codality as fixed effects and participant and item as random intercepts) using the *brms* package for R (Bürkner, 2017). As prior distributions, we chose normal (0, 1) for the parameter classes b, intercept, and exponential (10) for SD (Huff et al., 2025). Those prior distributions are weakly informative and constrain the parameter space by giving a low probability to extreme values. First, we compare the main effects model (including the main effects only) to the full model (including the interaction) using BF, thus explicitly testing for the interaction term. Running 20 unseeded computations of both models, the mean BF is 5.34 (range: 5.20–5.41). This can be considered substantial evidence favoring the main effect model, consequently assuming no interaction between education and codality. We further compared the main effects model against a model that only included codality as a main effect. The mean BF of this codality-only model over the main effects model was 11.24 (range: 11.02–11.54), thus providing strong evidence for the codality-only model and absence of education effects, consequently.

### Narrative comprehension, age and codality

#### Frequentist analysis

To study the effect of age, we fitted the second model with the fixed effects for age (at the timepoint of comprehension data collection; centered), codality, and their interaction, which were added stepwise. We preregistered to control for mental disorders (e.g., depression); however, due to the absence of clinically relevant levels of depression, we ultimately did not implement it. To ensure robust convergence, we used the “bobyqa” optimizer and increased the maximum number of iterations to 100,000 (glmerControl(optimizer = “bobyqa”, optCtrl = list(maxfun = 100000))). Convergence was confirmed using the check_convergence() function from the *performance* package, which returned no warnings and a gradient far below the threshold (e.g., gradient = 1.7e-06 for the main model in Table 2).

**Table 2:** Summary of the linear mixed model for the effects of age × codality

	Narrative comprehension		
*Predictors*	Odds ratios	CI	*p*
(Intercept)	3.70	2.59, 5.27	<.001
Age	1.03	0.99, 1.07	.139
Codality [picture]	1.80	1.06, 3.05	.030
Age Centered × codality [picture]	0.92	0.87, 0.98	.007

We expected narrative comprehension to decrease with higher age (H2). In contrast to Hypothesis 2, adding the *age* effect to the intercept model did not increase model fit, narrative comprehension did not decrease with higher age, χ^2^(2) = 0.1, *p* = .751.

**Fig. 3:**
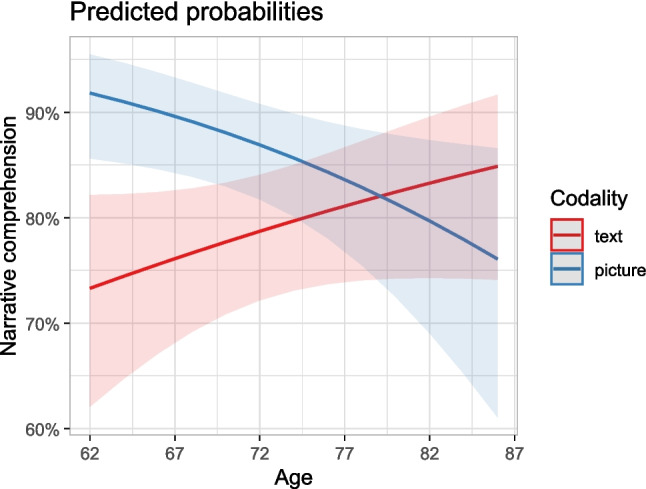
Predicted probabilities from the interaction model for the age and codality groups

We expected narrative comprehension to be higher for pictorial than for textual narratives independent of education or age (no interaction of education and age effects with story codality; H3). Supporting Hypothesis 3, adding the *codality* effect to the model containing age increased model fit, our findings revealed a significant difference in the comprehension of textual and pictorial narratives, *χ*^2^(1) = 4.25, *p* = .039. Pictorial narratives were generally comprehended better than textual ones. However, we also found an interaction effect between *age* and *codality*, χ^2^(1) = 7.18, *p* = .007, model fit increased through adding the interaction. This interaction indicates that with increasing age, the comprehension of pictorial narratives declined while the comprehension of textual narratives improved (see Fig. 3 and Table 2).

#### Bayesian analysis

To complement the results of the frequentist analysis, we again used Bayesian analyses. The methods were similar to the education analysis. The mean BF of the full model over the main effect model was 3.58 (range: 3.52–3.64), thus providing substantial evidence for the interaction of age and codality.

### Exploratory analysis

For the MoCA analysis and for the Memory Total Score, one more participant was excluded because of missing data. In an exploratory analysis, we examined the association between cognitive skills and narrative comprehension. For each fixed effect (CERAD-plus, MoCA, Memory Total Score, Memory Index), we fitted separate generalized linear mixed-effects models for the dependent measure narrative comprehension and random intercepts for participant and story with the “glmer” function of the *lme4* package (Bates et al., 2015) and conducted stepwise model comparisons for each of the cognitive tests. We started with an intercept model and added the respective cognitive skills variable followed by the codality effect and their interaction. We controlled for age at the timepoint of comprehension data collection (centered) and gender. The initial analysis indicated no significant influence of cognitive skills. Adding the CERAD-plus test score to the intercept model did not improve the model fit, χ^2^(1) = 2.16, *p =* .141, and the same was true for the MoCA test, χ^2^(1) = 0.75, *p =* .387. Adding the *codality* effect to the intercept model with CERAD-plus results increased model fit; our findings revealed a beneficial effect of pictorial codality, which led to better comprehension scores than textual codality, *χ*^2^(1) = 4.13, *p* = .042. Adding the *codality* effect to the intercept model with MoCA results increased model fit; our findings revealed a beneficial effect of pictorial codality, which led to better comprehension scores than textual codality, *χ*^2^(1) = 4.44, *p* = .035. Adding interaction did not improve model fits for both, CERAD-plus, χ^2^(1) = 2.11, *p* = .146 and MoCA test χ^2^(1) = 0.03, *p* = .871.

**Fig. 4:**
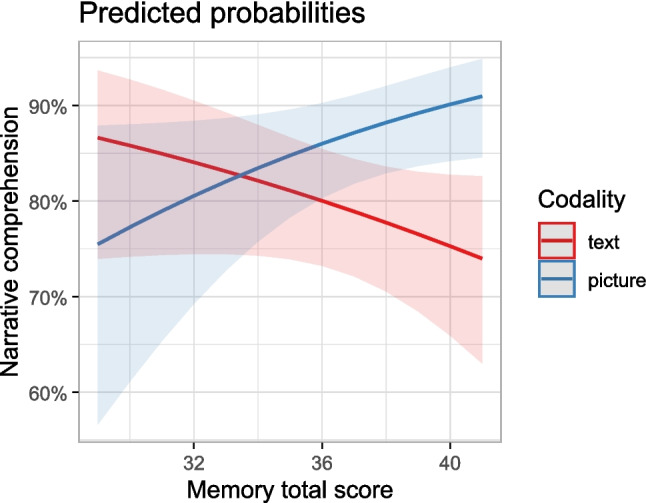
Predicted probabilities from the interaction model for the Memory Total Score and codality groups

Subsequently, we conducted a nuanced analysis using two composite memory scores from the CERAD – namely, the Memory Total Score and Memory Index, following Paajanen et al. (2014). This analysis revealed a significant interaction between the Memory Total Score and codality, χ^2^(1) = 6.30, *p* = .012, and Memory Index and codality, χ^2^(1) = 6.78, *p* = .009. Notably, participants with higher scores on the Memory Total Score demonstrated improved comprehension of visual narratives but decreased comprehension of textual narratives compared with participants with lower Memory Total Scores (see Fig. 4). The same pattern was visible for the memory index analysis, and that is why we provided only the plot for Memory Total Score. For the CERAD-plus test and MoCA test, we found no interaction effect with codality.

## Discussion

In the present study, we endeavored to illuminate the underexplored area of visual narrative comprehension in older adults, measured by an inference generation test in different codalities (textual and pictorial), and to examine how it connects to the age-related declines in memory and cognition. We investigated these questions using our original data (i.e., narrative comprehension) and data from the TREND longitudinal study (i.e., cognitive abilities, risk, and preventive factors). Our results shed light on four key findings related to the comprehension of narratives, including textual and visual media, in older adults.

First, narrative comprehension appeared to be unaffected by varying education levels in our study, disproving H1. The absence of education effects on comprehension can be attributed to the consistently high education level found within the current sample. We speculate that those participants had an average to above-average IQ and reading skills. This view is supported by a recent study, which found, using a more heterogeneous, representative study, that higher education levels are associated with higher narrative comprehension (Huff et al., 2025). The material used in the current study may be perceived as overly simple for the tested sample, potentially resulting in a reduced variance in narrative comprehension. Also, the low number of items could potentially reduce variance in the tested variable. Moreover, the MAIN test (Gagarina et al., 2019) was originally designed for children. To address this issue, we have developed a new set of narratives consisting of 24 stories (parallel in textual and pictorial version) to use in future studies. This will ensure sufficient variance and robust assessment of performance in bridging inference generation measurement.

Secondly, we found that cognitive skills and memory declined as age increased in the tested older adults, which is consistent with the findings of a vast number of earlier studies (e.g., Deary et al., 2009; Murman, 2015). Nevertheless, our findings indicated that contrary to H2, narrative comprehension did not decrease with advancing age. This finding aligns with previous results of a representative German study where age also did not affect narrative comprehension (Huff et al., 2025). It also supports the idea of Ramscar and colleagues (2014), who suggested that reduced performance in cognitive tests in older sample might be better explained by the heightened complexity and demands of memory search, stemming from the accumulation of knowledge over time and not by the cognitive decline itself. Taken together, our results show that narrative comprehension, as measured by the inference test, remains stable even if the same comprehenders show a decline in general cognitive skills and memory. However, previous research has consistently demonstrated that individuals with lower working memory capacity are less likely to generate more complex inferences (Linderholm, 2002; Rai et al., 2011; St George et al., 1997). This suggests that the degree of inferential complexity – or how long key information needs to be actively maintained in working memory – may determine the extent to which working memory is required to generate an inference (Hutson et al., 2021). In our study, the inferences may have been too simple to impose significant demands on working memory. Consequently, working memory appeared less critical for completing the task on simple inference recognition, allowing participants to perform successfully. Future studies should assess more complex inferences using more sophisticated materials. The materials we used were initially developed for children and are relatively short, which may not have sufficiently challenged working memory.

The lack of differences in narrative comprehension across different age groups of older participants in our study, despite declining memory and cognitive skills, suggests that existing schemas and accumulated knowledge (e.g., from reading) rooted in experience may play a more significant role in narrative comprehension than memory and cognitive processes. This aligns with the results from textual narratives, where it was observed that processes related to the situation model of comprehension for texts appear to remain relatively stable, even among older age groups (for an overview, see Radvansky & Dijkstra, 2007) and results from the audio narratives, where comprehension skill was also found to remain preserved (Schneider et al., 2000; Sommers et al., 2011). Crystallized intelligence strengthens as comprehenders grow older and gather additional knowledge and insight. It has been suggested that this intelligence improves as individuals age (Barbey, 2018). Moreover, higher age groups may compensate for the narrative comprehension difficulties with a high degree of previous experience and cognitive expertise, derived from cultural influence and education (Brandão & Parente, 2001). The stability of narrative comprehension in older individuals may also be attributed to their enjoyment of reading (Bouchard Ryan et al., 2003). Reading is a valuable means to enhance well-being, stay informed, fight feelings of isolation, and overcome other challenges commonly associated with aging (Ryan, 2019). A large UK study found that the enjoyment of reading in upper primary pupils had significantly fallen over the years (Sainsbury & Schagen, 2004), while in an older sample, regular reading habit was an extremely important part of their lives and the successful aging process (Bouchard Ryan et al., 2003).

The presentation method might also have affected the outcomes of this experiment. In particular, the simultaneous presentation of the beginning and end state pictures with the blank panel in the middle might have made the missing parts salient. This may have led the comprehenders to actively engage in bridging inference generation processes by activating front-end search mechanisms, as described by Hutson et al. (2018). Front-end search processes might lead comprehenders to seek content in the end-state picture that would help them in the generation of a bridging inference. For example, comprehenders make more fixations to gather the information necessary for making the inference (Hutson et al., 2018). This may account for the relatively high accuracy observed in the comprehension task. Future research should study the influence of the presentation format – including condition without a blank panel or with each part displayed on separate pages – on narrative comprehension. To cover finer differences, it would also benefit future studies to employ diverse methods for assessing inference generation. For example, it may be interesting to include questions on content and open-ended questions or to measure the reaction times. It seems likely that older adults can accurately judge the correctness of a suggested bridging inference, but whether they differ from the younger group in in the speed of forming this decision remains an open question. Measuring additionally the reaction times of responses may provide a more sensitive test of inference-making. Further studies would also benefit from including a broader spectrum of participants, particularly younger samples, and testing similar variables in both younger and older adults.

Thirdly, we expected narrative comprehension to be higher for pictorial than for textual narratives independent of education or age (no interaction of education and age effects with story codality; H3). Our findings support the idea of pictorial advantage, demonstrating better comprehension skills for pictorial narratives compared with textual ones. This finding aligns with previous research conducted on a representative German sample, which showed that pictorial narratives are generally better understood than textual ones across all demographics (Huff et al., 2025). Some researchers suggest that pictorial information may encourage more heuristic and automatic processing compared with textual information (Rudski & Volksdorf, 2002). However, we found that the comprehension of pictorial narratives tended to decrease, and comprehension of textual narratives tended to increase with higher age. Huff et al. (2025) suggested differences in the execution of similar processes for different codalities rather than fundamentally different processes. For example, our results suggest that processes such as laying a foundation, mapping, and shifting, as proposed in the structure-building framework (Gernsbacher, 1997; Gernsbacher et al., 1990), may involve varying levels of effort when applied to picture narratives compared with text narratives across different age groups. Moreover, it is well known that attentional control declines with age (Bier et al., 2017; Milham et al., 2002). We assume that the structure of text may be more beneficial in terms of attentional control. Textual forms of presentation may provide the needed basis for additional control that is lacking in the pictorial version of the stories. This is especially true for complex, nonschematic pictures. Also, regular reading habits in older adults may explain the fact that textual comprehension skill increases, especially in older samples, while comprehension of pictorial stories decreases.

From a cognitive perspective, there is also evidence supporting our findings. Specifically, it is not necessarily true that age-related perceptional difficulties will make older readers bad comprehenders. For example, Wang and colleague (2018) found in their eye-tracking study that Chinese readers of higher age (61–94 years) compensate for age-related reading difficulties by a more careful strategy (i.e., they do not skip words frequently, move forward more cautiously, and refixate words more often). While Chinese is a nonalphabetic language with a distinct structure (for more details, see Wang et al., 2018), readers of alphabetic languages (e.g., English or German) compensate for these difficulties by applying more risky reading strategies. They may skip words and are more likely to rely on prior context and only partial word information when inferring the meaning (Rayner, 2009; Rayner et al., 2006, 2013; Wang et al., 2018). One thing is clear, older adult readers in different languages have similar age-related reading difficulties, but they compensate for these difficulties and usually enjoy reading, which can explain the fact that especially textual comprehension gets better at a higher age. We can speculate that while pictorial codality is generally beneficial for narrative comprehension (Huff et al., 2025), the perception of multiple colorful pictures with intricate details within a brief timeframe, and the subsequent memorization of them, may pose challenges for older adults and require more effort in the older sample for processes like laying a foundation, mapping, and shifting, as proposed in the structure-building framework. This is because they typically lean towards reading activities. In contrast, younger adults, who are accustomed to constant exposure to visual information through extensive use of social media, perform processes such as laying a foundation, mapping, and shifting better when dealing with pictorial codality as opposed to textual formats. One important consideration is that the inferences presented for evaluation – whether correct or false – were consistently in text format, even when the story itself was presented pictorially. This was due to our utilization of standardized pictorial material, which could not be altered, while it was more feasible to adapt the textual version of inferences. However, this introduces a possible difference between the conditions. Essentially, in the condition where text is used to present the story, there is a match between the codality of presentation and the modality of inference generation measurement whereas in the case where pictures are used to present the story, there is a mismatch. This aspect should be taken into account in future studies examining various modalities.

Finally, our explorative finding of an interaction between memory scale (derived from the CERAD-plus test battery) and codality suggests that individuals with good memory skills benefit the most from using pictures. Magliano et al. (2016) showed that visuospatial and linguistic working memory systems support inference generation in visual narratives and play an important role in this process. We speculate that it might have been particularly challenging for participants with average or even low memory skills to concentrate and process the pictorial details, which often contain a large amount of information that needs to be processed simultaneously. Pictorial material may offer less inherent structure, making comprehension more difficult for older adults. In contrast, participants with above-average memory skills had no problems processing a large number of pictorial details simultaneously.

### Outlook

Our findings show that against the finding of a general decline in memory and cognitive scores with increasing age, the inferencing skill remains unaffected in older individuals. Although we can say that older participants are efficient in bridging different parts of a story, there are still several particularities in visual narrative comprehension in an older sample. Particularly, comprehension of pictorial narratives tends to decrease, and comprehension of textual narratives tends to increase with age. Also, comprehension of pictorial narratives is positively related to memory in our study. Exploratively, we found that inferencing skill for narratives is also unaffected by different preventive and risk factors. This aspect should be considered when aiming for effective information communication across all age groups and various social groups within the population.

## Supplementary Information

Below is the link to the electronic supplementary material.

Supplementary file1 (DOCX 1166 KB)

## Data Availability

All data are available on the project’s Open Science Framework page (https://osf.io/2zyuv/). We present an example of the study materials (see Fig. 1A). The license of the underlying material prevents its publication in this study as it is part of a diagnostic tool (MAIN–Multilingual Assessment Instrument for Narratives; Gagarina et al., 2019; https://main.leibniz-zas.de/). Nevertheless, all visual materials employed in this study can be retrieved from the online (https://main.leibniz-zas.de). Access to these materials requires prior acceptance of the corresponding copyright and license agreement.

## Footnotes

1 In this project, we differentiate between modality and codality. According to Clark and Salomon (1986), modality refers to the sensory path through which incoming information is perceived and processed. On the other hand, codality describes how the received information is encoded, it can be symbolic, pictorial, or verbal (Clark & Salomon, 1986; Huff et al., 2020).

## Code availability

Data and syntaxes used to conduct analyses in a statistical package R are openly available at the project’s Open Science Framework page (https://osf.io/2zyuv/).
