# Supplementary material for: Inference generation in older adults: Comparing pictorial and textual comprehension in the context of cognitive decline
Source: Mem Cognit. 2025 Jul 18;54(1):27–44. doi: 10.3758/s13421-025-01736-7 (PMC12864196; doi:10.3758/s13421-025-01736-7)
Supplement: Supplementary file 1 — Supplementary file1 (DOCX 1166 KB) [file 13421_2025_1736_MOESM1_ESM.docx]

**Appendix A:**  **Descriptive data for risk and preventive factors**

**Table 1** *Descriptive data for risk and preventive factors*

| Factor | Mean / Distribution | Range | Cut-offs |
| --- | --- | --- | --- |
| Depression (BDI-II) (Beck et al., 1996; Hautzinger et al., 2009). | M = 6 (SD = 6.92) | 0-41 points | A total score of 0-8 points reflects no evidence of depression, 9-13 points indicate minimal depression, 14-19 points indicate mild depression, 20 points or more indicates moderate depression and 29 points or more indicates severe depression. A maximum of 63 points can be achieved. |
| Anxiety doctoral assessment and self-assessment. | 0: n = 128 participants  1: n = 8 participants | 0 (no) or 1 (yes) | Participants marked 'yes' (1) or 'no' (0) to indicate the presence of anxiety disorder based on both the doctor's assessment and their own self-assessment. |
| Chronic pain doctoral assessment. | 0: n = 119 participants  1: n = 17 participants | 0 (no) or 1 (yes) | Participants marked 'yes' or 'no' to indicate the presence of chronic pain based on the doctor's assessment. |
| Chronic pain self-assessment. | 0: n = 121 participants  1: n = 15 participants | 0 (no) or 1 (yes) | Participants marked 'yes' or 'no' to indicate the presence of chronic pain based on their own self-assessment. |
| Long-term stress (Perceived Stress Scale; Cohen et al., 1983). | M = 11.8 (SD = 6.42) | 0 to 40 | Higher scores reflect  greater levels of stress. |
| Sleep quality (Item 16 from the BDI-II) | 0: n = 78 participants  1: n = 27 participants  2: n = 20 participants  3: n = 3 participants  4: n = 2 participant  5: n = 0 participants  6: n = 6 participants | 0 to 6 | Item 16 from BDI-II has levels 0 (my sleeping habits have not changed), 1 (I sleep a little more/less than usual), 2 (I sleep a lot more/ less than usual), and 3 (I sleep most of the day/ I wake up 1-2 hours earlier than usual and then I can't go back to sleep) which are coded like this in our data: 0=0, 1 = 1a, 2 = 1b, 3 = 2a, 4 = 2b, 5 = 3a, 6 = 3b. |
| Physical activity (frequency of sport; (Thefeld et al., 1999) | 1: n = 10 participants  2: n = 13 participants  3: n = 32 participants  4: n = 37 participant  5: n = 44 participants | 1 to 5 | Participants answered a question about the overall frequency of sports activities (5-point scale from “>4 h /week” = 5, to “no sport at all” = 1) |
| Mental fitness (frequency) | 1: n = 2 participants  2: n = 5 participants  3: n = 13 participants  4: n = 36 participant  5: n = 80 participants | 1 to 5 | Participants answered a question about the overall frequency of mental fitness activities (5-point scale from “>4 h /week” = 5 to “no mental fitness activities at all” = 1). |
| Stable social contacts (companionship) (six-item loneliness scale; Gierveld & Tilburg, 2006). | 0: n = 88 participants  1: n = 24 participants  2: n = 8 participants  3: n = 5 participants  4: n = 1 participant  5: n = 6 participants  6: n = 4 participants | 0 to 6 points | The lower the value, the lower the loneliness experienced by the individual and thus, the higher the companionship experienced by the individual. |

**Appendix B: Risk and preventive factors for narrative comprehension**

**Risk and preventive factors for narrative comprehension**

The supplement answers the question of whether physical and mental activity, as well as stable social contacts (companionship), act as possible preventive factors for narrative comprehension and whether depression, anxiety disorder, chronic pain, poor sleep quality, and stress act as risk factors. Based on the reviewed literature, we derived two hypotheses (preregistered here: https://osf.io/4kf3e?view_only=6248cb53a51242e48a9c6774def2d40e). We expected narrative comprehension to decrease with higher depression and anxiety scores, with diagnosed chronic pain, long-term stress, and bad sleep quality (H4), and narrative comprehension to increase with higher physical activity, higher mental fitness, and stable social contacts (companionship) (H5).

*Preventive and Risk Factors for Narrative Comprehension in Older Age*

We calculated separate correlations between the factor age (at the timepoint of latest TREND data collection for each variable) and each cognitive, risk, and preventive factor. We observed a statistically significant increase in the frequency of physical activity with increasing age, *r =* .25, *t*(134) = 3.02, *p* = .003 (see Figure 2). However, for other risk and preventive factors, we did not find any significant correlations with age. The levels of depression, *r* = .15, *t*(134) = 1.8, *p* = .074, anxiety, *r* = -.15, *t*(134) = -1.72, *p* = .088, and chronic pain *r* = -.04, *t*(134) = -.52, *p* =.603 (see Figure 1), did not demonstrate statistically significant changes with increasing age. Sleep quality did not significantly change with increasing age, *r* = -.02, *t*(134) = -.19, *p* = .849, and long-term stress did not significantly change with increasing age, *r* = -.05, *t*(134) = -0.55, *p* = .58 (see Figure 1). In our analysis of preventive factors, only engagement in sports exhibited a statistically significant association with age. Other preventive factors did not significantly change with increasing age. The frequency of mental fitness did not significantly change with increasing age, *r* = -.07, *t*(134) = -.82, *p* = .416; we assessed companionship using the reversed the six-item loneliness scale (the lower the value, the higher companionship) (Gierveld & Tilburg, 2006) and also did not significantly change with increasing age, *r =* .06, *t*(134) = -.69, *p* = .49 (see Figure 2).

**Figure 1**

*Correlations between age and risk factors (depression, anxiety, chronic pain, long term stress, and bad sleep quality)*


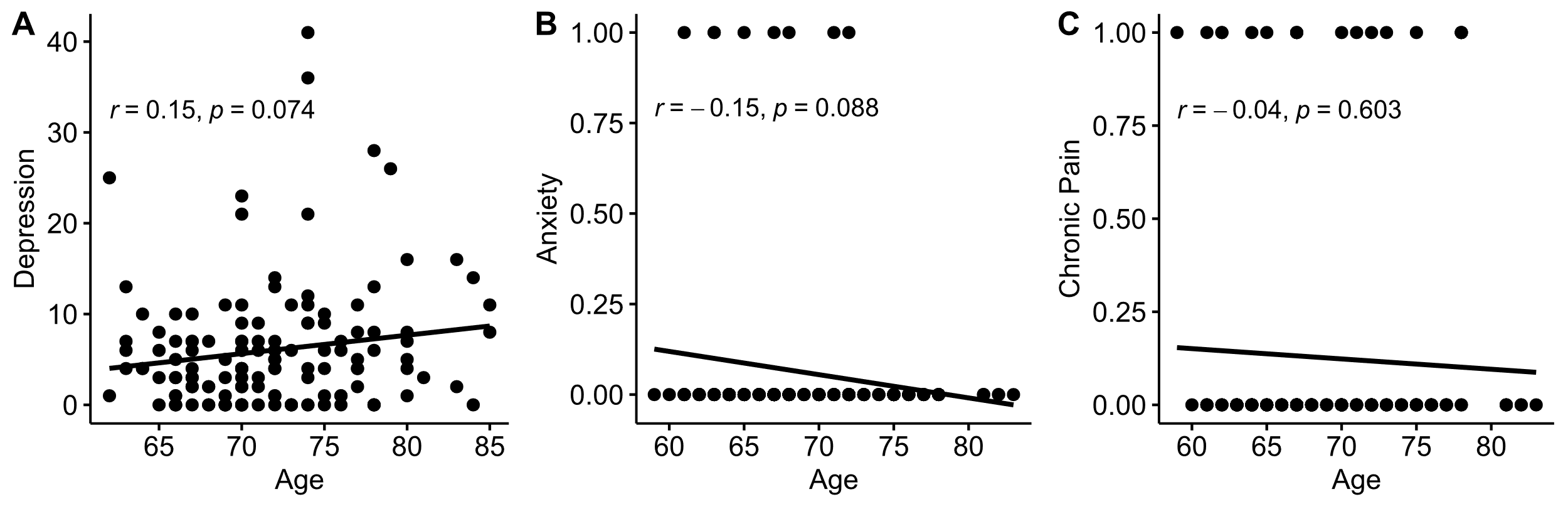


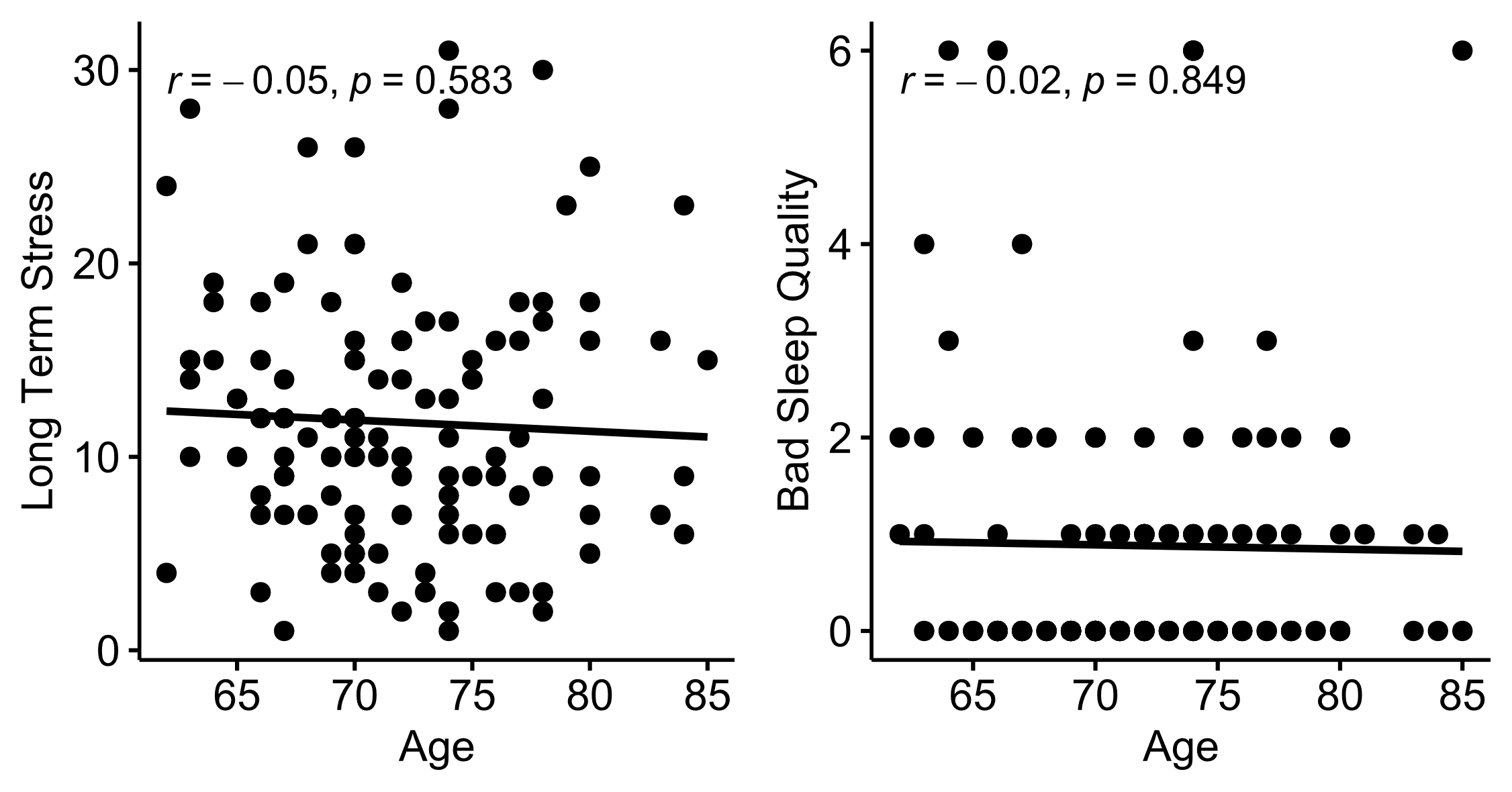


**Figure 2***Correlations between age and preventive factors (sport, mental fitness, and companionship)*

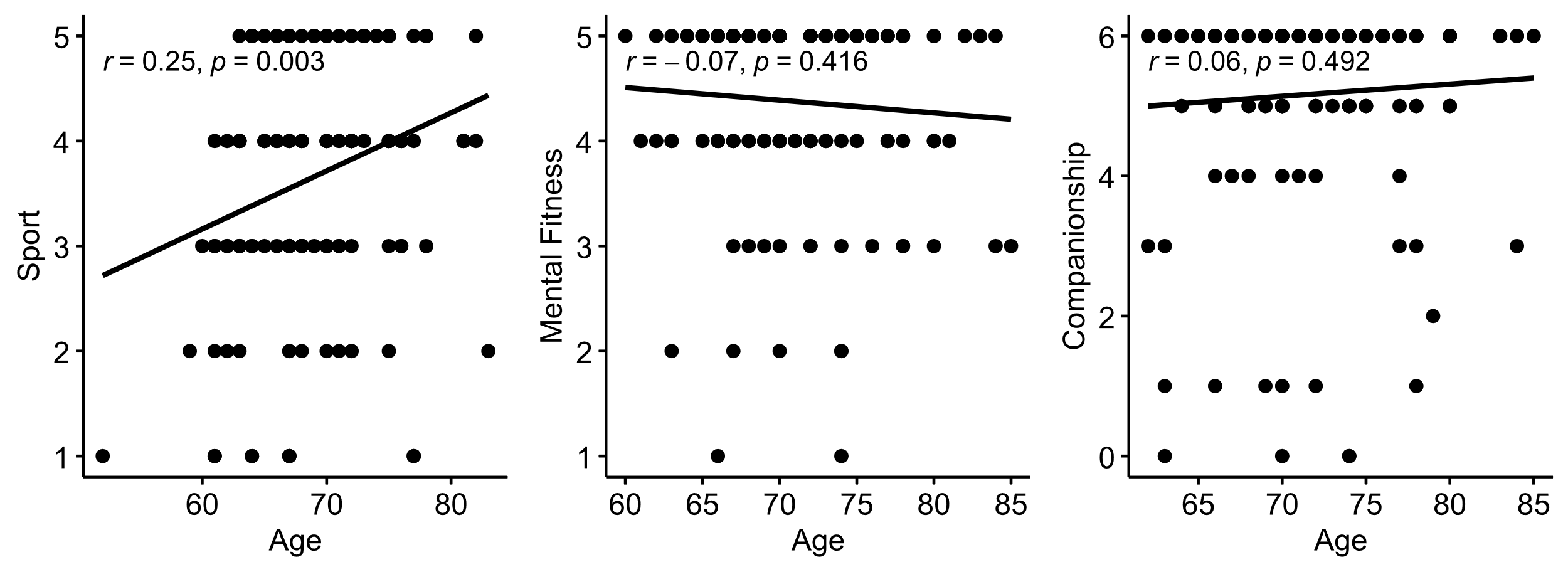


**Risk factors for narrative comprehension**

For hypothesis 4, we expected narrative comprehension to decrease with higher depression and anxiety scores, with diagnosed chronic pain, long-term stress, and bad sleep quality (H4). A table containing descriptive data for each factor will be presented in Appendix A. Starting from an intercept model with participant and item as random intercepts, we added the fixed effect of each risk factor (depression, anxiety, chronic pain, long-term stress, and sleep quality) separately and compared the models accordingly. We controlled for age at the timepoint of comprehension data collection (centered) and gender. Inconsistent with Hypothesis 4, narrative comprehension did not decrease with higher depression and anxiety scores, with diagnosed chronic pain, long-term stress, or with bad sleep quality.

We conducted stepwise model comparisons of generalized linear mixed models, and the analysis revealed no significant effect of depression, *X*^2^(1) = 1.19, *p =* .276 (see Table 1).

**Table 1** *Summary of the linear mixed model for the effect of depression.*

|  | **Narrative comprehension** | | |
| --- | --- | --- | --- |
| *Predictors* | *Odds Ratios* | *CI* | *p* |
| (Intercept) | 4.41 | 3.12 – 6.24 | <.001 |
| Depression | 1.01 | 0.99 – 1.04 | .283 |
| age | 0.99 | 0.96 – 1.02 | .594 |
| gender [female] | 1.06 | 0.76 – 1.47 | .737 |

The analysis of anxiety, as a second possible risk factor, also revealed no significant connection to narrative comprehension for both doctoral diagnosis, *X*^2^(1) = 0.52, *p =* .473, (Table 2) and self-assessment, *X*^2^(1) = 0.26, *p =* .872, (see Table 3).

**Table 2** *Summary of the linear mixed model for the effect of diagnosed anxiety disorder.*

|  | **Narrative comprehension** | | |
| --- | --- | --- | --- |
| *Predictors* | *Odds Ratios* | *CI* | *p* |
| (Intercept) | 4.65 | 3.35 – 6.46 | <.001 |
| diagnosed anxiety disorder | 1.31 | 0.62 – 2.74 | .480 |
| Age | 1.00 | 0.97 – 1.03 | .818 |
| gender [female] | 1.09 | 0.79 – 1.51 | .591 |

**Table 3** *Summary of the linear mixed model for the effect of self-diagnosed anxiety.*

|  | **Narrative comprehension** | | |
| --- | --- | --- | --- |
| *Predictors* | *Odds Ratios* | *CI* | *p* |
| (Intercept) | 4.70 | 3.39 – 6.52 | <.001 |
| anxiety self-assessment | 1.06 | 0.53 – 2.13 | .872 |
| age | 1.00 | 0.97 – 1.03 | .746 |
| gender [female] | 1.09 | 0.79 – 1.52 | .591 |

The analysis for diagnosed chronic pain also revealed no significant connection to narrative comprehension, *X*^2^(1) = 0.97, *p =* .325, (Table 4) the same was true for self-assessment of chronic pain, *X*^2^ (1) = 1.03, *p =* .309, (Table 5).

**Table 4** *Summary of the linear mixed model for the effect of diagnosed chronic pain.*

|  | **Narrative comprehension** | | |
| --- | --- | --- | --- |
| *Predictors* | *Odds Ratios* | *CI* | *p* |
| (Intercept) | 4.81 | 3.46 – 6.68 | <.001 |
| diagnosed chronic pain | 0.79 | 0.50 – 1.25 | .316 |
| age | 0.99 | 0.96 – 1.02 | .703 |
| gender [female] | 1.13 | 0.81 – 1.56 | .475 |

**Table 5** *Summary of the linear mixed model for the effect of self-diagnosed chronic pain.*

|  | **Narrative comprehension** | | |
| --- | --- | --- | --- |
| *Predictors* | *Odds Ratios* | *CI* | *p* |
| (Intercept) | 4.82 | 3.46 – 6.71 | <.001 |
| chronic pain self-assessment | 0.77 | 0.48 – 1.25 | .299 |
| age | 1.00 | 0.97 – 1.03 | .815 |
| gender [female] | 1.11 | 0.81 – 1.54 | .508 |

No significant results were found for long-term stress, *X*^2^(1) = 0.33, *p =* .566, (Table 6) and sleep quality, *X*^2^(2) = 0.01, *p =* .920 (Table 7).

**Table 6** *Summary of the linear mixed model for the effect of long-term stress*

|  | **Narrative comprehension** | | |
| --- | --- | --- | --- |
| *Predictors* | *Odds Ratios* | *CI* | *p* |
| (Intercept) | 5.08 | 3.34 – 7.73 | <.001 |
| long-term stress | 0.99 | 0.97 – 1.02 | .563 |
| age | 0.99 | 0.97 – 1.02 | .734 |
| gender [female] | 1.14 | 0.81 – 1.60 | .460 |

**Table 7** *Summary of the linear mixed model for the effect of sleep quality*

|  | **Narrative comprehension** | | |
| --- | --- | --- | --- |
| *Predictors* | *Odds Ratios* | *CI* | *p* |
| (Intercept) | 4.69 | 3.35 – 6.56 | <.001 |
| strong changes in sleeping pattern | 1.01 | 0.90 – 1.13 | .919 |
| age | 0.99 | 0.97 – 1.03 | .734 |
| gender [female] | 1.10 | 0.79 – 1.52 | .573 |

**Preventive factors for narrative comprehension**

We expected narrative comprehension to increase with higher physical activity, higher mental fitness, and stable social contacts (companionship) (H5). A table containing descriptive data for each factor will be presented in Appendix A. The same procedure from the risk factors was applied to the protective factors for narrative comprehension. We conducted again stepwise model comparisons of generalized linear mixed models with the same structure for all protective factors and the analysis revealed no significant effect for any of the tested positive factors. Not confirming Hypothesis 5, narrative comprehension did not increase with higher physical activity, higher mental fitness, and stable social contacts (companionship).

The amount of physical activity had no significant effect on narrative comprehension, *X*^2^(1) = 0.05, *p =* .821, (Table 8) the same was true for mental fitness, *X*^2^(1) = 0.02, *p =* .885 (Table 9).

**Table 8** *Summary of the linear mixed model for the effect of physical activity*

|  | **Narrative comprehension** | | |
| --- | --- | --- | --- |
| *Predictors* | *Odds Ratios* | *CI* | *p* |
| (Intercept) | 5.00 | 2.70 – 9.26 | <.001 |
| frequency of physical activity | 0.98 | 0.86 – 1.13 | .820 |
| age | 1.00 | 0.97 – 1.03 | .774 |
| gender [female] | 1.09 | 0.79 – 1.51 | .598 |

**Table 9** *Summary of the linear mixed model for the effect of mental fitness*

|  | **Narrative comprehension** | | |
| --- | --- | --- | --- |
| *Predictors* | *Odds Ratios* | *CI* | *p* |
| (Intercept) | 4.44 | 1.89 – 10.4 | .001 |
| frequency of mental fitness | 1.01 | 0.85 – 1.21 | .884 |
| age | 0.99 | 0.97 – 1.03 | .736 |
| gender [female] | 1.10 | 0.80 – 1.52 | .552 |

The next possible protective factor that we analyzed was stable social connections (companionship), for which we also found no significant connection to narrative comprehension, *X*^2^(1) = 1.89, *p =* .17, (Table 10).

**Table 10** *Summary of the linear mixed model for the effect of stable social connections*

|  | **Narrative comprehension** | | |
| --- | --- | --- | --- |
| *Predictors* | *Odds Ratios* | *CI* | *p* |
| (Intercept) | 4.48 | 3.21 – 6.25 | <.001 |
| six item loneliness scale loneliness score | 1.08 | 0.96 – 1.21 | .179 |
| age | 1.00 | 0.97 – 1.03 | .779 |
| gender [female] | 1.07 | 0.77 – 1.48 | .685 |

**Appendix C: Bayesian analyses**

**Bayesian analyses**

Bayesian statistics enables explicit testing of the null hypothesis, complementing the frequentist approach effectively. We followed Jeffreys’ (1961) heuristic: Bayes Factors (BF) between 1-3 are anecdotal, 3-10 substantial, 10-30 strong, 30-100 very strong, and BFs >100 extreme evidence for the alternative hypothesis. Fractions (1/3, 1/10, etc.) represent evidence strength for the null hypothesis.

All models included 1000 warm-up iterations with a total of 40000 Markov Chain Monte Carlo (MCMC) draws. Four cores were run in parallel, resulting in four separate MCMC chains.

All $\hat{R}$-values were under 1.01, which is necessary but not sufficient to confirm model convergence (Vehtari et al., 2021). Therefore, we additionally performed visual posterior predictive checks to ensure that the models accurately predicted the data (Gabry et al., 2019). See Figure 1 for the narrative comprehension and education analyses and Figure 2 for the narrative comprehension and age analyses, respectively.

**Hypotheses**

- H1: Narrative comprehension increases with higher education.
- H2: Narrative comprehension decreases with higher age.
- H3: Narrative comprehension to be higher for pictorial than for textual narratives independent of education or age (no interaction of education and age effects with story codality).

**Narrative comprehension and education**

We used Bayesian analyses to complement the results of the frequentist analysis. We fitted the same models (including education and codality as fixed effects and participant and item as random intercepts) using the brms-package for R (Bürkner, 2017). As prior distributions, we chose *normal(0,1)* for the parameter classes b, Intercept, and *exponential(10)* for SD (Huff et al., 2023). Those prior distributions are weakly informative and constrain the parameter space by giving a low probability to extreme values. First, we compare the main effects model (including the main effects only) to the full model (including the interaction) using BF, thus explicitly testing for the interaction term. Running 20 unseeded computations of both models, the mean BF is 5.34 (range = 5.20 - 5.41). This can be considered substantial evidence favoring the main effect model, consequently assuming no interaction between education and codality. We further compared the main effects model against a model that only included codality as a main effect. The mean BF of this codality-only model over the main effects model was 11.24 (range = 11.02 - 11.54), thus providing strong evidence for the codality-only model and absence of education effects, consequently (see Table 1).

***Table 1*.**

*Model* summary of the main effects model

**Group-Level Effects**

| Group | Parameter | Estimate | Est. Error | l-95% CI | u-95% CI | $\hat{R}$ | Bulk_ESS | Tail_ESS |
| --- | --- | --- | --- | --- | --- | --- | --- | --- |
| clip_id | sd(Intercept) | 0.43 | 0.13 | 0.18 | 0.69 | 1.00 | 11525 | 7521 |
| part_id | sd(Intercept) | 0.07 | 0.06 | 0.00 | 0.22 | 1.00 | 25489 | 19404 |

**Population-Level Effects**

| Parameter | Estimate | Est. Error | l-95% CI | u-95% CI | $\hat{R}$ | Bulk_ESS | Tail_ESS |
| --- | --- | --- | --- | --- | --- | --- | --- |
| Intercept | 1.29 | 0.17 | 0.97 | 1.63 | 1.00 | 29549 | 27688 |
| codality_d1 | 0.53 | 0.24 | 0.06 | 1.01 | 1.00 | 30821 | 27220 |

**Notes**

- Draws were sampled using sampling(NUTS).
- For each parameter, Bulk_ESS and Tail_ESS are effective sample size measures.
- $\hat{R}$ is the potential scale reduction factor on split chains (at convergence, $\hat{R}$ = 1).

**Model Information**

- Family: Bernoulli
- Links: mu = logit
- Formula: NC_cor ~ codality_d + (1 | part_id) + (1 | clip_id)
- Data: dat05 (Number of observations: 1088)
- Draws: 4 chains, each with iter = 10000; warmup = 1000; thin = 1; total post-warmup draws = 36000

***Figure 1*.**

*Posterior predictive check of the resulting main effects model (y: observed data; yrep: predictive distribution).*

**
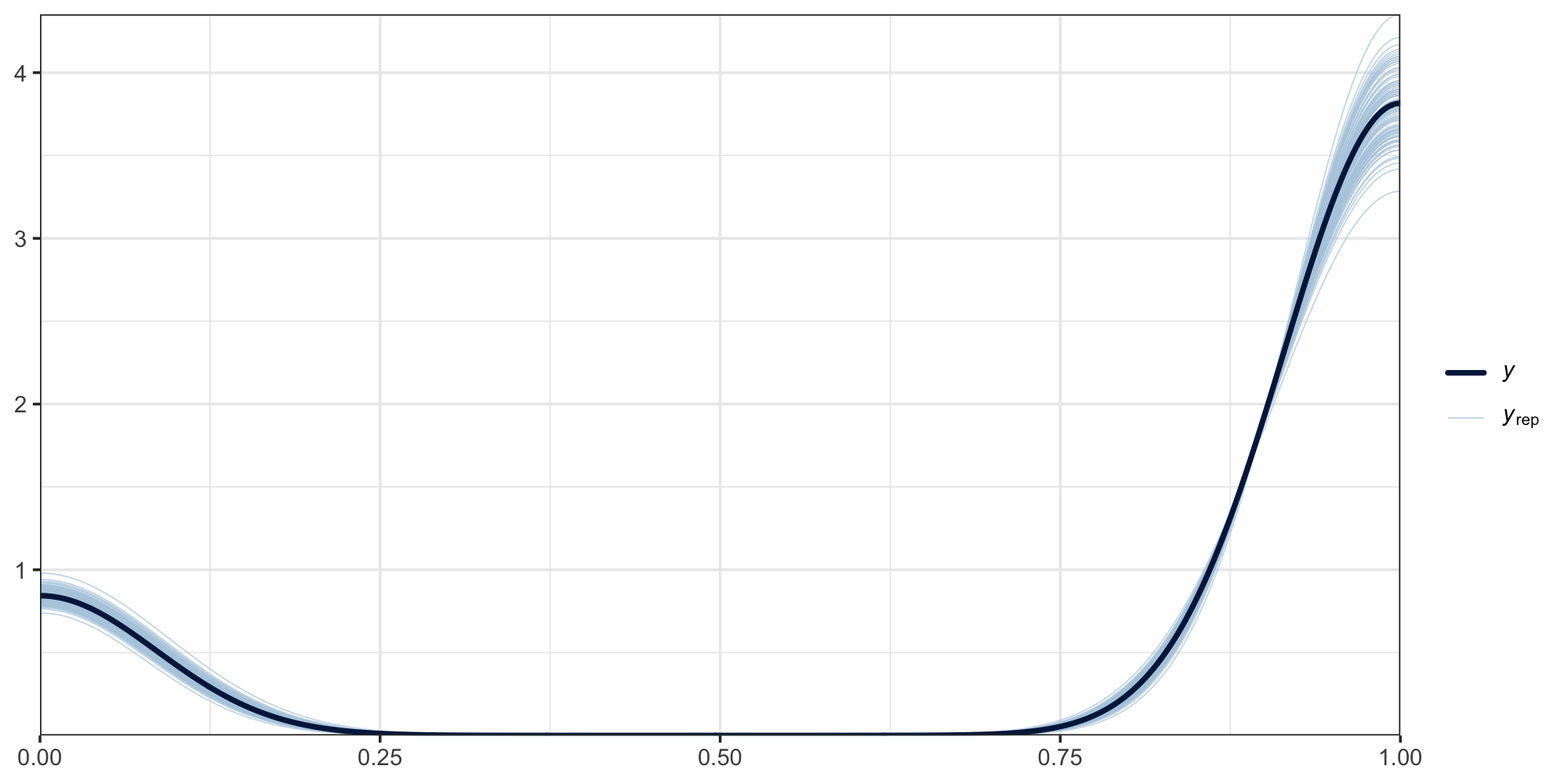
**

**Narrative comprehension and age**

To complement the results of the frequentist analysis, we again used Bayesian analyses. The methods were similar to the education analysis. The mean BF of the full model over the main effect model was 3.58 (range = 3.52 - 3.64), thus providing substantial evidence for the interaction of age and codality, see Table 2). Table 2 summarizes the full model.

***Table 2*.**

*Model summary of the full model.*

**Group-Level Effects**

| Group | Parameter | Estimate | Est. Error | l-95% CI | u-95% CI | $\hat{R}$ | Bulk_ESS | Tail_ESS |
| --- | --- | --- | --- | --- | --- | --- | --- | --- |
| clip_id | sd(Intercept) | 0.63 | 0.16 | 0.35 | 0.99 | 1.00 | 13556 | 21781 |
| part_id | sd(Intercept) | 0.14 | 0.10 | 0.01 | 0.37 | 1.00 | 15369 | 18017 |

**Population-Level Effects**

| Parameter | Estimate | Est. Error | l-95% CI | u-95% CI | $\hat{R}$ | Bulk_ESS | Tail_ESS |
| --- | --- | --- | --- | --- | --- | --- | --- |
| Intercept | 1.32 | 0.22 | 0.89 | 1.76 | 1.00 | 16580 | 22073 |
| age_c | 0.03 | 0.02 | -0.01 | 0.07 | 1.00 | 38249 | 26529 |
| codality_d1 | 0.63 | 0.32 | 0.01 | 1.27 | 1.00 | 17752 | 22558 |
| age_c:codality_d1 | -0.09 | 0.03 | -0.15 | -0.02 | 1.00 | 37606 | 27915 |

**Notes**

- Draws were sampled using sampling(NUTS).
- For each parameter, Bulk_ESS and Tail_ESS are effective sample size measures.
- $\hat{R}$ is the potential scale reduction factor on split chains (at convergence, $\hat{R}$ = 1).

**Model Information**

- Family: Bernoulli
- Links: mu = logit - Formula: NC_cor ~ age_c * codality_d + (1 | part_id) + (1 | clip_id)
- Data: dat05 (Number of observations: 1088)
- Draws: 4 chains, each with iter = 10000; warmup = 1000; thin = 1; total post-warmup draws = 36000

***Figure 2*.**

*Posterior predictive check of the resulting full model (y: observed data; yrep: predictive distribution).*

**
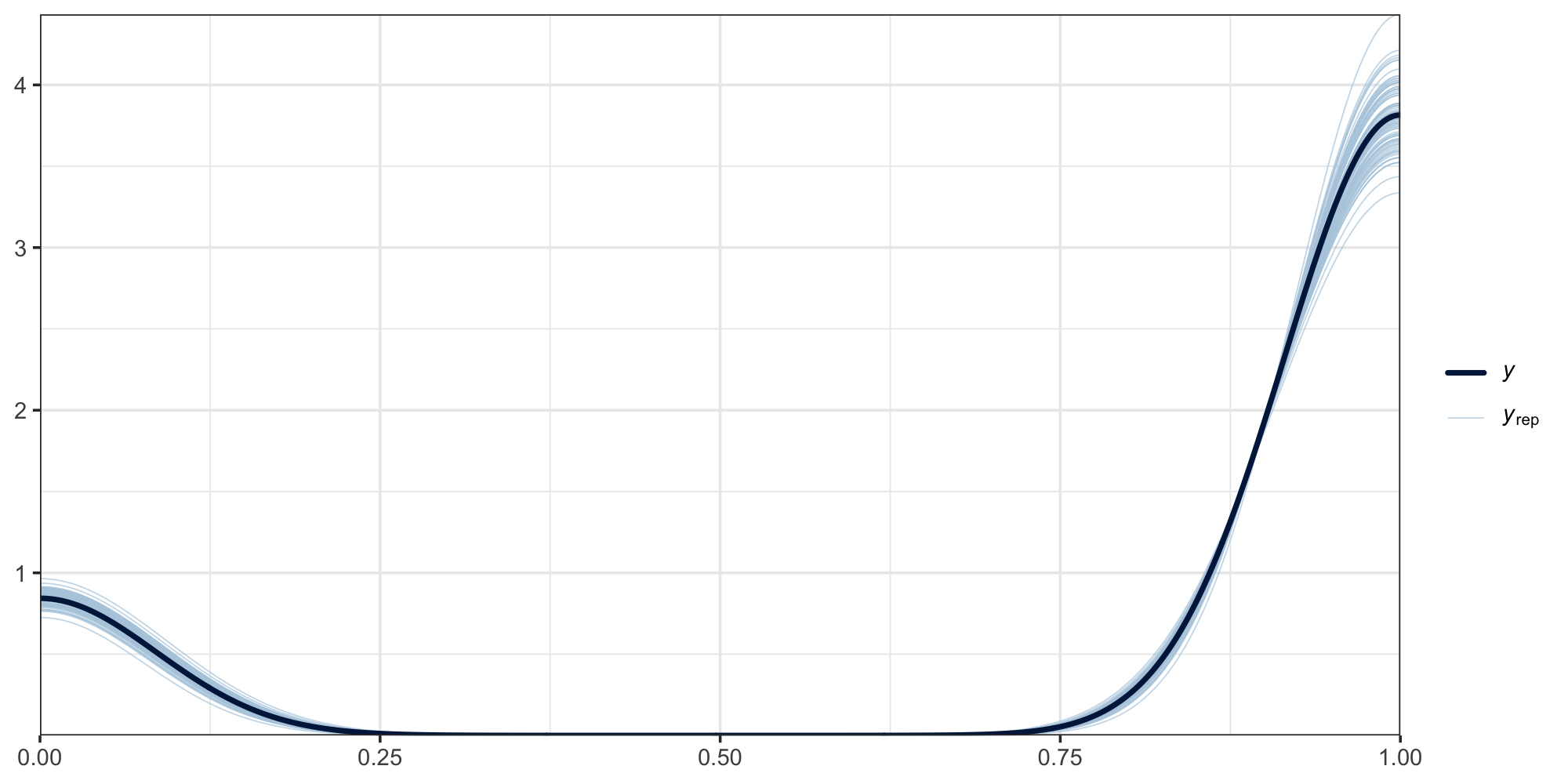
**

**References**

Bürkner, P.-C. (2017). brms: An R Package for Bayesian Multilevel Models Using Stan. *Journal of Statistical Software*, *80*, 1–28. <https://doi.org/10.18637/jss.v080.i01>

Gabry, J., Simpson, D., Vehtari, A., Betancourt, M., & Gelman, A. (2019). Visualization in Bayesian Workflow. *Journal of the Royal Statistical Society Series A: Statistics in Society*, *182*(2), 389–402. <https://doi.org/10.1111/rssa.12378>

Jeffreys, H. (1961). *Theory of Probability.* (3rd ed.). Clarendon Press.

Vehtari, A., Gelman, A., Simpson, D., Carpenter, B., & Bürkner, P.-C. (2021). Rank-Normalization, Folding, and Localization: An Improved Rˆ for Assessing Convergence of MCMC (with Discussion). *Bayesian Analysis*, *16*(2), 667–718. <https://doi.org/10.1214/20-BA1221>
